# Highly efficient construction of infectious viroid-derived clones

**DOI:** 10.1186/s13007-019-0470-4

**Published:** 2019-08-01

**Authors:** Joan Marquez-Molins, Jose Antonio Navarro, Vicente Pallas, Gustavo Gomez

**Affiliations:** 1 Institute for Integrative Systems Biology (I2SysBio), Consejo Superior de Investigaciones Científicas (CSIC), Universitat de València (UV), Parc Científic, Cat. Agustín Escardino 9, 46980 Paterna, Spain; 2 Instituto de Biología Molecular y Celular de Plantas (IBMCP), Consejo Superior de Investigaciones Científicas (CSIC), Universitat Politècnica de València, CPI 8E, Av. de los Naranjos s/n, 46022 Valencia, Spain

**Keywords:** Viroid, Cloning, Dimers, IIs enzymes, Agro-infiltration

## Abstract

**Background:**

Viroid research generally relies on infectious cDNA clones that consist of dimers of the entire viroid sequence. At present, those dimers are generated by self-ligation of monomeric cDNA, a strategy that presents several disadvantages: (i) low efficiency, (ii) it is a non-oriented reaction requiring tedious screenings and (iii) additional steps are required for cloning into a binary vector for agroinfiltration or for in vitro RNA production.

**Results:**

We have developed a novel strategy for simultaneous construction of a viroid dimeric cDNA and cloning into a multipurpose binary vector ready for agroinfiltration or in vitro transcription. The assembly is based on IIs restriction enzymes and positive selection and supposes a universal procedure for obtaining infectious clones of a viroid independently of its sequence, with a high efficiency. Thus, infectious clones of one viroid of each family were obtained and its infectivity was analyzed by molecular hybridization.

**Conclusion:**

This is a zero-background strategy for direct cloning into a binary vector, optimized for the generation of infectious viroids. As a result, this methodology constitutes a powerful tool for viroid research and exemplifies the applicability of type IIs restriction enzymes and the lethal gene *ccd*B to design efficient and affordable direct cloning approaches of PCR products into binary vectors.

**Electronic supplementary material:**

The online version of this article (10.1186/s13007-019-0470-4) contains supplementary material, which is available to authorized users.

## Background

Viroids are small single-stranded plant-pathogenic RNAs, being considered the smallest (246–401 nt) autonomous infectious nucleic acids known so far [1]. These pathogens replicate and move systemically in host plants causing phenotypic effects that range from severe symptoms to latent infections [2, 3]. More than 50 species of viroids have been described, being currently grouped into the families *Pospiviroidae* and *Avsunviroidae*, based on their replication site (nuclei and chloroplasts, respectively), presence of particular sequence domains, and properties of their infectious cycle [4]. In both groups, replication takes place through a rolling-circle mechanism. Host RNA polymerases transcribe longer-than-unit replication intermediates that are cleaved and ligated to form circular monomers [5]. In nuclear viroids, only the (+) RNA intermediate is processed, whereas in members of the *Avsunviroidae* family both (+) and (−) intermediates are self-cleaved by hammerhead ribozymes and ligated subsequently [6].

The particularities of viroids, such as being the smallest known pathogens, their extremely high mutation rate [7] or the fact that were the first circular RNAs discovered [8], highlight the importance of the research on this topic. In this regard, novel methods have been developed by working on viroids, resulting in important applications such as determination of RNA folding and base pairing networks or setting the basis of plasmid purification kits [8]. Nonetheless, the study of viroids might still help to elucidate key biological pathways in plants such as RNA traffic [9–11] or genetic regulation by epigenetic modifications [12–14].

The study of viroid replication and pathogenesis extensively relies on infectious cDNAs clones which consist of dimers of the entire viroid sequence whose transcripts mimic the longer-than-unit replication intermediates and therefore can be processed into unitary, circular RNAs within the cell [15, 16]. Due to this circular nature of viroidal genome, dimeric constructs are used in order to ensure that a full monomeric RNA can be produced independently of the cloning site. In this sense, experimental results indicate that viroid dimeric cDNAs always present a higher infectivity compared to monomeric cDNAs [15, 17]. Thus, viroid dimeric cDNAs can be used for in vitro transcription and subsequent plant inoculation. However, the dimeric cDNA can be more easily and efficiently delivered to plant cells by agrobacterium-mediated transient plant expression, which avoids the requirement of obtaining a sufficient quantity of viroid transcript in vitro, and therefore has been established as a more convenient strategy for inducing viroid-infection in diverse host plants [18–20].

The considerable decrease of the DNA synthesis price is supposing a revolution to the technology of recombinant DNA. However, the presence of repeated sequences is still an important limitation that often makes impossible the synthesis by commercial suppliers. Consequently, generating infectious viroids, which requires the dimerization of the sequence, and therefore intrinsically implies the generation of repetitions, is especially problematic.

At present, infectious dimeric viroids are constructed by self-ligation of monomeric cDNA in presence of T4 DNA ligase [15, 21, 22]. Dimeric head-to-tail clones on the desired (+) orientation are identified by restriction fragment analysis or using specific primers in colony PCR. However, the ratio of positive clones on the desired orientation could be scarce since self-ligation of blunt ends fragments is a non-controlled process in which only a small fraction forms an exact oriented dimer. Moreover, additional steps are required to introduce the dimer under an expression cassette in a binary vector for agroinfiltration or in a vector harboring T7/T3 polymerase promoters for in vitro transcription [18].

In order to avoid tedious screenings and reduce timework, we have developed an oriented assembly strategy for efficient generation of viroid dimeric cDNAs. Assembly of DNA parts was introduced by Gibson Assembly and has been adapted to several methods [23]. Gibson assembly relies on generating cohesive ends and can produce a seamless fusion if overlapping DNA fragments are used. Those extremes can be generated by exonuclease activity being filled the gaps by a DNA polymerase, or alternatively using type IIs restriction enzymes, such as *Bsa*I or *Bsm*BI, which have non-palindromic recognition sites that are distal from the cleavage site (N1/N5) [24]. Taking advantage of this property, we have constructed dimeric infectious constructs of one viroid of each family developing a novel strategy of simultaneous dimerization and cloning into a binary vector specifically designed for *Agrobacterium*-mediated viroid-inoculation.

## Methods

### Binary vector construction

A binary vector suitable for the direct cloning and transcription/expression of dimeric viroid was engineered from the binary vector pMDC32B-AtMIR390a-Bc [25]. This vector was digested with restriction enzymes *Eco*RI and *Hin*dIII (Thermo Scientific™, Waltham, MA, USA), and the resulting fragment of 8.9 kb was excised from a 1% agarose gel and purified using GeneJET Gel Extraction Kit (Thermo Scientific™, Waltham, MA, USA). All reactions were performed following manufacturer’s instructions. DNA fragments were amplified with PrimeSTAR™ HS DNA Polymerase (Takara, Kusatsu, Japan) and ligations were set with an insert:vector ratio of 3:1 and 3U of T4 DNA ligase (Promega, Madison, WI, USA) being transformed into DB3.1 *Escherichia coli* cells. In order to obtain a positive selection vector, the lethal gene *ccd*B was cloned into an engineered pSK vector designed for cloning, after digestion with *Nco*I and *Nhe*I, a sequence between a duplicated promoter CaMV 35S and the Potato Protease inhibitor II terminator (PoPit) [26]. This vector was modified to eliminate a *Hin*dIII recognition after the PoPit terminator, by amplifying the plasmid with inverted oligonucleotides: Fw mut-*Hin*dIII and Rv mut-*Hin*dIII. The lethal gene *ccd*B was amplified from pMDC32B-AtMIR390a-Bc using Fw ccdB-*Nco*I/b and Rv ccdB-*Nhe*I/b (designed to generate compatible ends with *Nco*I and *Nhe*I) and ligated to the aforementioned pSK vector after *Bsa*I digestion (Thermo Scientific™, Waltham, MA, USA). Additionally, 2×35S:ccdB:PoPit cassette was amplified with Fw T7-35S *Hin*dIII, to introduce the T7 RNA polymerase promoter, and Rv M13, digested with *Eco*RI and *Hin*dIII (Thermo Scientific™, Waltham, MA, USA) and ligated to the above mentioned backbone of pMDC32. The resulting vector was named pMD201t, number 201 indicates 2×35S:*ccd*B:PoPit, respectively, and the letter t makes references to the T7 promoter. All oligonucleotides used for generating this construct are listed in Additional file 1: Table S1.

### Dimeric viroid cDNA construction

Monomeric form of Hop Stun Viroid (HSVd) (AN Y09352.1), previously cloned in a pBluescript II SK vector [27], was used as a template to generate the two DNA fragments required for the dimer assembly. PCR reactions were performed with the following reaction mixture: 1.25U PrimeSTAR™ HS DNA Polymerase (Takara, Kusatsu, Japan), 5 μL of 5× Buffer, 2 μL of 2.5 mM dNTP mixture, 25 µM of each primer, 75 ng of plasmid template and sterilized water up to 25 µL. PCR conditions were 30 cycles of 10 s at 98 °C, 5 s at 55 °C and 21 s at 72 °C. One reaction was performed with the primers Fw D1-HSVd and Rv D2-HSVd, and the other with Fw D3-HSVd and Rv D4-HSVd (Additional file 1: Table S1). These DNA fragments were purified together using GeneJET Gel Extraction Kit (Thermo Scientific™, Waltham, MA, USA) and a one-pot reaction was set as follows: 10 U of *Bsa*I (Thermo Scientific™, Waltham, MA, USA), 3U T4 DNA ligase (Promega, Madison, WI, USA), 1 µL of ligase buffer 10× (Promega, Madison, Wi, USA), 50 ng of pMD201t and 300 ng of the digested DNA fragments in a final volume of 10 µL. The incubation was performed using a thermocycler with the following conditions: an initial step of 20 min at 37 °C, 20 cycles of 1 min at 37 °C and 4 min a 16 °C, finally holding on the temperature at 16 °C until transformation.

Monomeric form of ELVd cDNA (genebank AJ536613) was synthetized as gBlocks^®^ (Integrated DNA Technologies Inc., Coralvelle, IA, USA) and used as a template, as described above, for PCR amplification with Fw D1-ELVd Rv D2-ELVd and Fw D3-ELVd Rv D4-ELVd. PCR conditions were identical to those aforementioned except that 50 ng of the gene fragment was used for each reaction as template. PCR products were purified together using GeneJET Gel Extraction Kit (Thermo Scientific™, Waltham, MA, USA) and digested with 10 U of *Bsm*BI (Thermo Scientific™, Waltham, MA, USA) according to manufacturer instructions in a final volume of 40 µL, and eventually purified and concentrated into a final volume of 20 µL using GeneJET Gel Extraction Kit (Thermo Scientific™, Waltham, MA, USA). Finally, a ligation reaction was set with: 3U T4 DNA ligase (Promega, Madison, Wi, USA), 1 µL of ligase buffer 10× (Promega, Madison, WI, USA), 50 ng of *Bsa*I digested pMD201t and 300 ng of the digested monomer fragments in a final volume of 10 µL. This ligation was incubated for 2 h at room temperature.

1–2 µL of the HSVd/ELVd cDNAs ligation to pMD201t, respectively, was transformed into DH5-Alpha electro competent cells and plated onto kanamycin agar plates (Additional file 2: Figure S1). 8 colonies were analyzed by PCR colony using Go-Taq (Promega, Madison, WI, USA) and oligonucleotides Fw 35S-AMV and Rv Popit (Additional file 2: Figure S2). Plasmid extraction was performed with GeneJET Plasmid Miniprep Kit (Thermo Scientific™, Waltham, MA, USA) and resulting constructs were sequenced using Rv Popit. The oligonucleotides used are listed in Additional file 1: Table S1.

### Viroid inoculation

Cotyledons of *Cucumis sativus* cv. Marketer and *Solanum melongena* cv. Black Beaut*y* were agro-infiltrated with a culture of *A. tumefaciens* strain C58 harbouring the correspondent binary vector, pMD201t-HSVd for *C. sativus* and pMD201t-ELVd for *S. melongena*. The overnight grown bacterial culture was diluted in infiltration buffer (MES 0.1 M, MgCl_2_ 1 M) up to an optical density at 600 nm of 1 and injected on the abaxial side of one cotyledon using a needle-less syringe. Plants were kept in a photoperiod of 16 h under visible light and 30 °C (light)/25 °C (darkness) for *C. sativus* and 25 °C (light)/18 °C (darkness) for *S. melongena*. Samples of systemic leaf tissue were collected at 21 and 28 days after viroid inoculation.

### In vitro transcription

Viroid transcripts were generated by transcription of 400 ng of linearized pMD201t HSVd/ELVd (Digested with *Eco*RI) with T7 RNA polymerase (Takara, Kusatsu, Japan) for 3 h according to manufacturer instructions. 1 μL of each 10 μL reaction was loaded into a sterile 1% agarose gel, by serial dilutions (0.1, 0.3 and 0.6 μL). RiboRuler High Range RNA Ladder (Thermo Scientific™, Waltham, MA, USA) was used to estimate the RNA concentration; as for the loaded volume (0.83 μL) each ladder band corresponds to 50 ng.

### RNA extraction and northern/dot blot

Total RNA was extracted from systemic leaves as described previously [26]. 2–5 μg of total RNA per sample were mixed with solid urea and then loaded into a PAGE 5% UREA 8 M and TBE 89 mM gel. RNA electrophoresis was performed at 200 V for 1 h and then RNA was transferred to a nylon membrane using MiniProtean 3 system (BioRad, Hercules, CA, USA). Transference conditions were 100 V for 1 h at 4 °C in TBE buffer 1×. Nucleic acids transferred to the membrane (northern) or directly applied onto the nylon membrane (dot, 1 μL of total RNA per sample) were covalently fixed by using ultraviolet light (700 × 100 J/cm^2^). Hybridisation and chemiluminescent detection were performed as previously described [28].

## Results

### Dimeric clones construction

**Fig. 1:**
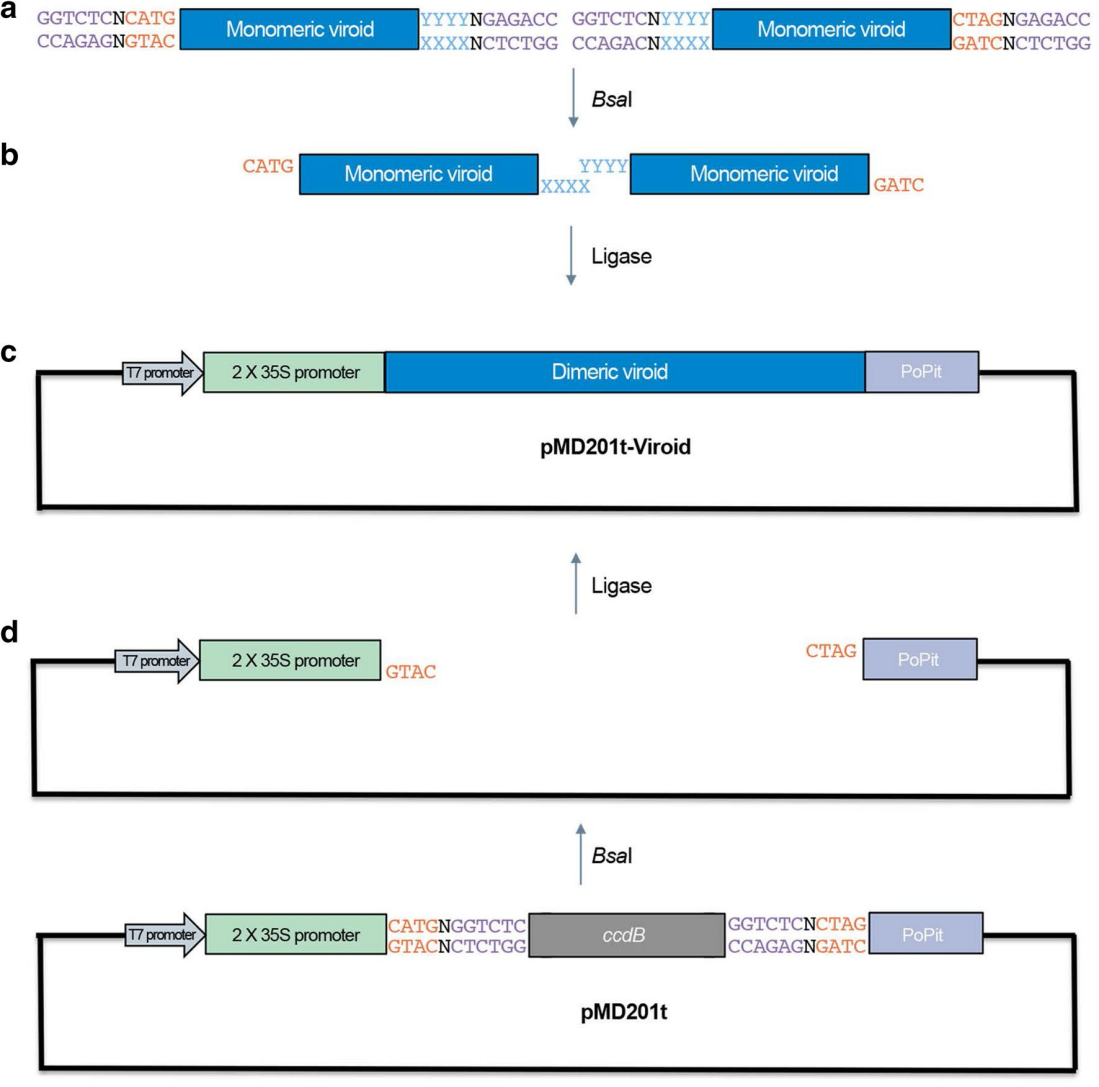
Schematic representation of viroid dimerization and subsequent assembly in a binary vector. **a** Two different pairs of primers are used to generate distal *Bsa*I recognition sites (magenta) in such a way that compatible ends for assembly can be obtained. **b** In a single reaction of simultaneous restriction and ligation the two viroid monomers (blue) are ligated between them and to a binary vector with compatible cohesive ends (orange). **c** Specifically, the viroid dimer is inserted into an expression cassette that contains and a duplicated 35S, a constitutive promoter for plant expression and a PoPit terminator. Additionally, this binary vector has a T7 promoter for in vitro transcription. The dimeric viroid cDNA sequence replaces a lethal gene ccdB, thus guaranteeing an optimal efficiency of the reaction. **d** Detail of the generation of the receptor–vector starting from pMD201t construct)

In order to enable the direct cloning of viroid dimeric cDNAs, a suitable vector for generating transcripts was specifically designed. This multipurpose vector includes a T7 RNA polymerase promoter for in vitro transcription and a double constitutive promoter 35S for agrobacterium-mediated transient plant transformation (Fig. 1). The location of the T7 promoter, upstream the 35S, generates transcripts of the plus polarity of the viroid RNA (defined as the most abundant in vivo) which is the convenient to establish a viroid infection.

The resulting binary vector was named pMD201t because it was engineered from pMDC32B [25] and includes the same lethal gene (*ccd*B) for positive efficient selection. In pMD201t, *ccd*B is excised by *Bsa*I digestion, generating four nucleotides overhangs in each strand (GTAC in the negative strand and CTAG in the positive strand). The generation of these cohesive ends enables the orientated assembly of a desired cDNA designed to generate compatible ends upon digestion.

Experimental validation of the functionality of pMD201t was performed with a member of the *Pospiviroidae* family, Hop stunt viroid (HSVd), in which *Bsa*I is a non-cutter and with an *Avsunviroidae* member, Eggplant latent Viroid (ELVd), which contains a *Bsa*I recognition site, thus exemplifying the applicability of the strategy for obtaining infectious clones of phylogenetically unrelated viroids.

**Fig. 2:**
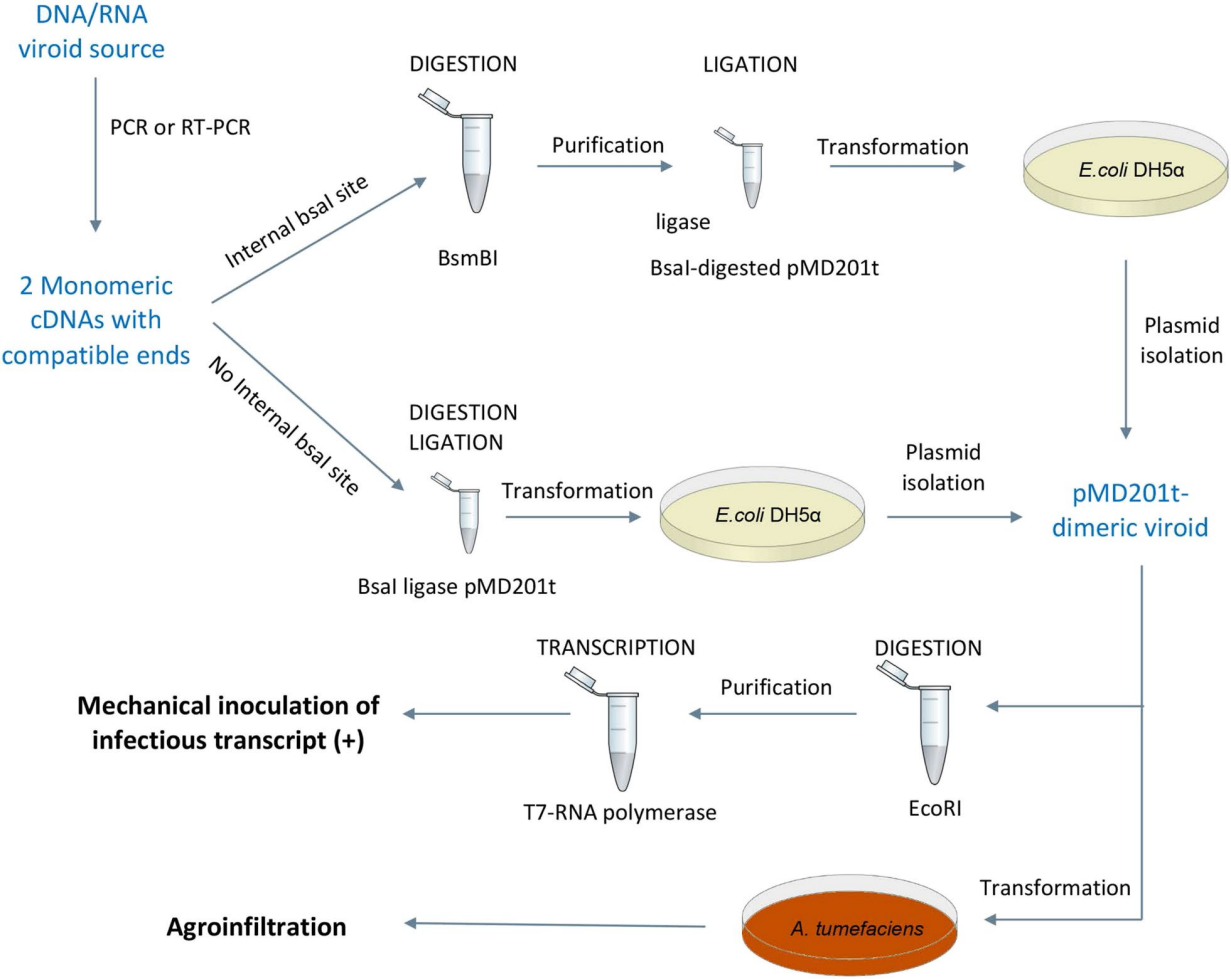
Workflow proposed to obtain infectious clones of a viroid. Viroid sequence can be amplified from infected tissue by RT-PCR or from a DNA source by PCR. If the viroid sequence does not contain a *bsa*I recognition site, the viroidal cDNA can be directly assembled into the binary vector (pMD201t), replacing a lethal gene, in a simultaneous *bsa*I restriction and ligation. Conversely, if there the viroid contains a *bsa*I recognition site, it can be cloned using another IIs enzyme, such as *Bsm*BI. Once digested and purified, the viroid cDNA is dimerized by ligation to a previously digested pMD201t. The dimeric viroidal cDNA cloned into pMD201t (pMD201t-viroid) can be used to generate the infectious RNA transcript in vitro, using T7 RNA polymerase onto a linearized plasmid (digested with *Eco*RI). Additionally, the pMD201t-viroid can be transformed into *Agrobacterium tumefaciens* for transient plant transformation and subsequent production of infective RNA in vivo

The dimeric cDNA of HSVd was obtained as depicted in Fig. 1. Briefly, the viroid monomer was amplified using two pairs of primers (Fw D1-HSVd/Rv D2-HSVd and Fw D3-HSVd/Rv D4-HSVd, Additional file 1: Table S1) designed to generate, after *Bsa*I digestion, cohesive ends that result in the merge of two monomers whose extremes are compatible with *Bsa*I-digested pMD201t. Therefore, a seamless fusion of the HSVd dimer to pMD201t can be generated (the resultant construct was denominated pMD201t-HSVd). Taking advantage of the fact that IIs enzymes are active in ligase buffer [24], a one-pot reaction of restriction/ligation was set (Fig. 2).

On the other hand, dimeric ELVd was generated following an equivalent strategy, except for the requirement of a previous step of digestion and purification prior to ligation to the binary vector pMD201t (Fig. 2). Primers (Fw D1-ELVd/Rv D2-ELVd and Fw D3-ELVd/Rv D4-ELVd, Additional file 1: Table S1) were designed analogously for amplifying the viroid monomer but containing *Bsm*BI recognition sites to produce the cohesive ends. After amplification and digestion with *Bsm*BI, DNA fragments were ligated to pMD201t (the obtained construct was denominated pMD201t-ELVd).

The resulting ligations were transformed into *ccd*B-sensitive *E. coli* cells (DH5 Alpha), which lack the gene *ccd*A for producing the antitoxin. As a consequence, plasmid molecules in which *ccd*B has not been replaced cannot propagate, which results in a zero-background positive selection (Additional file 2: Figure S1). In this regard, colony PCR was performed to validate the presence of the viroid cDNA dimer in eight colonies for each construct (Additional file 2: Figure S2).

### The dimeric viroid-derived clones are highly infectious

**Fig. 3:**
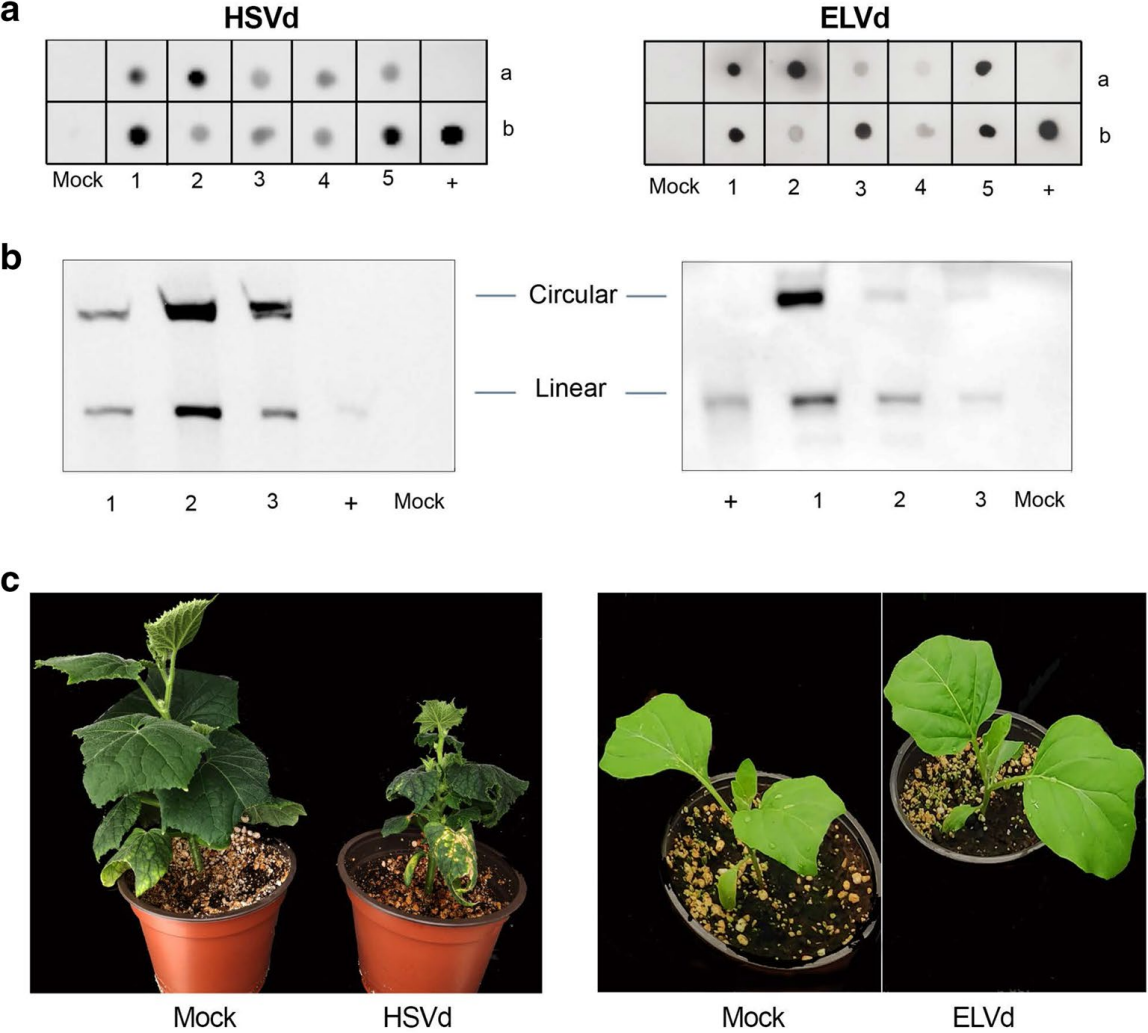
Infectivity of viroid constructs. **a** Dot blot of systemic leaves at 21 dpi of ten plants (a1–5/b1–5) agroinfiltrated with HSVd construct (left panel) and ELVd construct (right panel) or and two plants agroinfiltrated with empty vector (Mock a/b). HSVd and ELVd transcripts of plus polarity were used as positive control (+ b). **b** Northern blot of systemic leaves at 28 dpi of representative cucumber and eggplant plants agroinfiltrated with HSVd construct (1–3 left) and with ELVd construct (1–3 right) respectively. In both case, plants inoculated with empty vectors were used as mock control. Monomeric linear transcript of HSVd and ELVd, respectively were used as positive controls (+). **c** Figure showing representative symptomatic (cucumber-HSVd) and asymptomatic (eggplant-ELVd) infected plants at 28 dpi

Once the constructs were obtained and sequenced, agro-mediated inoculation bioassays were conducted to analyze the infectivity of the HSVd- and ELVd-derived clones. Cotyledons of ten cucumber plants were agroinfiltrated with pMD201t-HSVd as this wide-host range viroid produces characteristic symptoms in this experimental host [27]. All inoculated plants (10/10) were positive for HSVd detection (Fig. 3a left) and presented the characteristic symptoms of infection at 28 dpi, mainly characterized by growth delay and leaves dwarfism (Fig. 3c left). A northern blot was carried out to detect the mature forms of HSVd-RNA in systemic leaves of three representative infected-plants (Fig. 3b left). Related to ELVd-derived clone assay, eggplant cotyledons were agroinfiltrated with pMD201t-ELVd. In coincidence with the observed in HSVd-infection, hybridization assays evidenced that ELVd could be efficiently detected by dot blot in all inoculated plants at 28 dpi (10/10) (Fig. 3a right). Similarly, mature forms of ELVd were detected by northern blot in systemic leaves recovered from three representative infected-plants (Fig. 3b). As was previously described [29], ELVd infects eggplant without producing any visible symptom (Fig. 3c right).

Finally, to check the efficiency of the constructed dimeric clone as template for in vitro transcription assays (+) strand transcripts of the HSVd and ELVd were obtained using T7 RNA polymerase (Additional file 2: Figure S3).

## Discussion

We have developed an optimized strategy for oriented assembly of viroid-derived cDNA to generate dimeric infectious clones. The process consists of direct cloning into a binary vector that can be transformed into *Agrobacterium tumefaciens* to establish viroid infection by agroinfiltration or employed to generate RNA transcripts in vitro using T7 RNA polymerase.

Type IIs restriction enzymes *Bsa*I and *Bsm*BI were chosen because they cleave outside their recognition site at N1/N5 and thus these four nucleotides can be freely selected, being arranged in our strategy to produce a seamless fusion between the monomeric viroid cDNAs and to the binary vector pMD201t. Other IIs enzymes whose cleavage products result in four nucleotides overhangs exist, such as BtgZ1 (N10/N14), BveI (N4/N8) and BbS1 (N2/N6) but are less desirable because of the larger separation between the recognition and cleavage sites, requiring longer primers. In particular, the binary vector pMD201t was designed with *Bsa*I recognition sites and we propose the use of those for any compatible sequence because it is considerably more affordable than the other IIs restrictions enzymes; in fact, most of the type IIs-based assembly methods rely on this enzyme [30].

Considering the small size of viroids genome (246–401 nucleotides), the occurrence of both restriction sites (*Bsa*I and *Bsm*BI) in the same viroid is extremely unlikely-lower than 0.20% of the know viroid variants—(Additional file 3: Table S2), but in any case, could be overcome using the other aforementioned IIs restriction enzymes or by the assembly of the fractioned monomers produced by internal IIs enzyme cleavage. This later strategy would be feasible unless the four nucleotides left overhangs were compatible with the four chosen for dimerization or vector assembly, which is a very remote possibility. However, that approach will decrease the efficiency of ligation as the number of parts to be ligated is increased. These considerations clearly validate the universality of our strategy for constructing infectious clones of any viroid. Additionally, this strategy could be applied to expand the possibilities of site directed mutagenesis in viroids, by using mutagenic primers to amplify the monomeric sequence, which could be subsequently dimerized, therefore avoiding the apparition of unexpected mutations previously reported when direct mutagenesis of the dimeric viroid is used [31].

Nonetheless, we have developed this assembly strategy for obtaining dimers of viroids, but it can be repurposed for oriented seamless ligation of any sequence directly onto binary vectors with an optimal efficiency. Consequently, our approach illustrates the applicability of type IIs restriction enzymes and the lethal gene *ccd*B to design efficient and affordable cloning approaches into binary vectors.

## Conclusion

The purpose of this study was to develop a global (applicable to phylogenetically unrelated families), innovative, and quickly strategy to construct infectious viroid clones suitable to be employed in Agrobacterium-mediated inoculation and/or as template for in vitro transcription of viroid RNA. Our results support, that this methodology constitutes a valuable tool for viroid research and reinforce the applicability of type IIs restriction enzymes and the lethal gene ccdB to design efficient and direct cloning approaches of PCR products into binary vectors.

## Additional files

**Additional file 1.** Analisys of cloning efficience.

**Additional file 2.** Analisys of pMD201t constructs by colony-PCR.

**Additional file 3.** In vitro transcription of viroid RNA from pMD201t-HSVd and pMD201t-ELVd constructs.

## Data Availability

Not applicable.
