# Supplementary material for: Highly efficient construction of infectious viroid-derived clones
Source: Plant Methods. 2019 Aug 1;15:87. doi: 10.1186/s13007-019-0470-4 (PMC6670230; doi:10.1186/s13007-019-0470-4)
Supplement: Supplementary file 1 — Additional file 1. Analisys of cloning efficience. [file 13007_2019_470_MOESM1_ESM.pdf]

Additional file 1: Table S1 Detail of the oligos used in this work.

Oligonucleotide	Sequence 5'-3'
Fw D1-HSVd	GGTCTCCCATGGCTGGGGAATTCTCGAGTTG
Rv D2-HSVd	GGTCTCAAGGGGCTCAAGAGAGGAT
Fw D3-HSVd	GGTCTCCCCCTGCTGGGGAATTCTCGAGTTG
Rv D4-HSVd	GGTCTCTCTAGAAGGGGCTCAAGAGAGGAT
Fw D1-ELVd	GACGTCTCCCATGGGGTGGTGTGTGCCACCCCT
Rv D2-ELVd	GCGCCGTCTCCTATGGGGAGAGGTCGTCCTCTATC
Fw D3-ELVd	GCAGCGTCTCTCATAGGGTGGTGTGTGCCACCCCTG
Rv D4-ELVd	GCGCCGTCTCGCTAGCTATGGGGAGAGGTCGTCCTCTAT
Rv Popit	TGGATGATCTCTTTCTCTTATTCAG
Fw 35S-AMV	CATTTGGAGAGGGTTTTTATTTTT
Fw ccdB-NcoI/b	GCACCATGGGAGACCATTAGGCACCCCAGG
Rv ccdB-NheI/b	GCTGCTAGCGAGACCGTCGAGGTGCAGAC
Fw mut-HindIII	CCATATACAAGCcTTGATAATCGAATTCC
Rv mut-HindIII	GGAATTCGATTATCAAgGCTTGTATATGG
Fw T7-35S HindIII	CGAAAGCTTTAATACGACTCACTATAGGCTTACTCGAGCTCGGGCCCTCTACTCC
Rv M13	AGCGGATAACAATTTCACACAGG
