# Supplementary material for: Highly efficient construction of infectious viroid-derived clones
Source: Plant Methods. 2019 Aug 1;15:87. doi: 10.1186/s13007-019-0470-4 (PMC6670230; doi:10.1186/s13007-019-0470-4)
Supplement: Supplementary file 2 — Additional file 2. Analisys of pMD201t constructs by colony-PCR. [file 13007_2019_470_MOESM2_ESM.pdf]

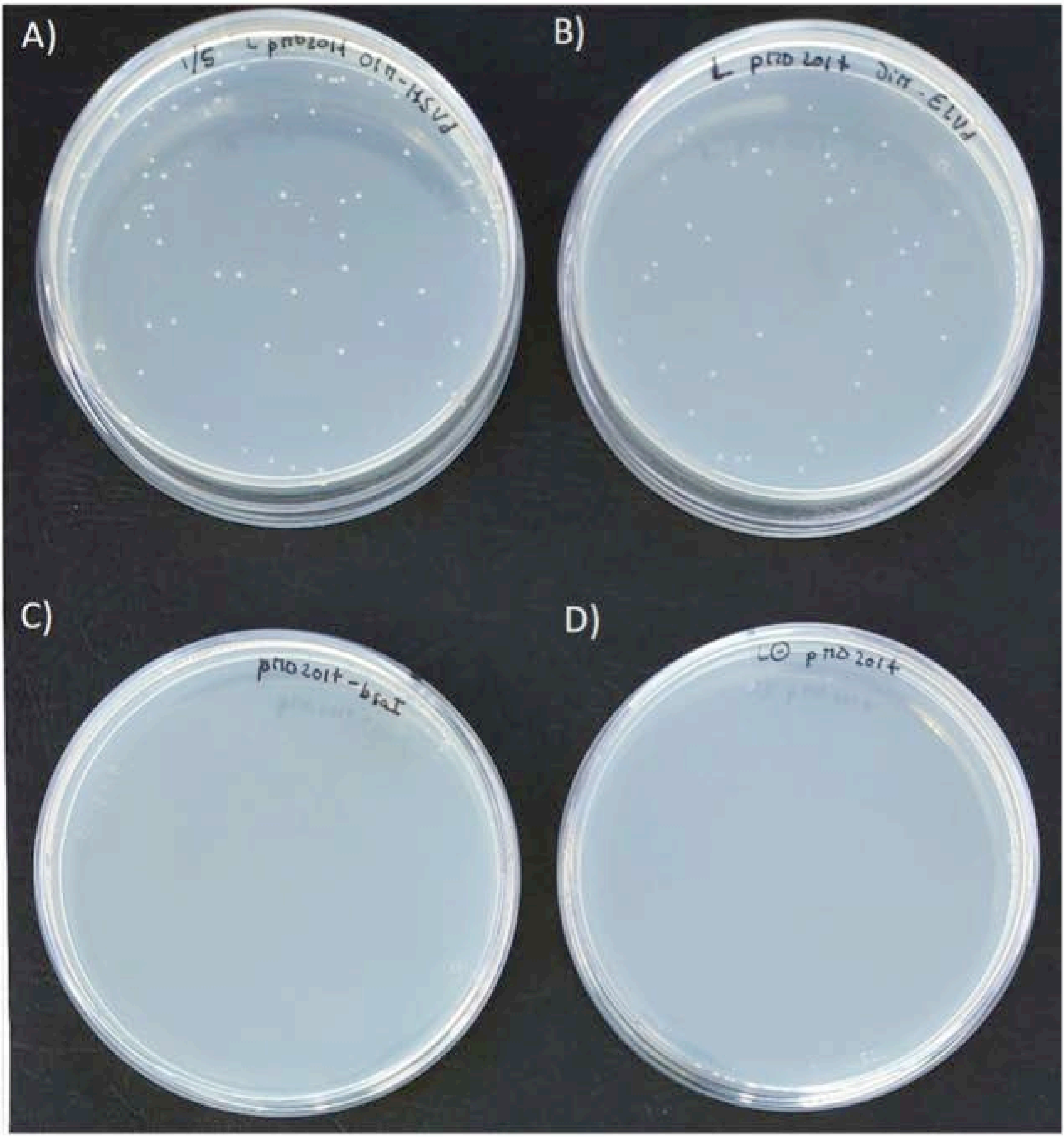

Additional file S1: Figure S1. A) Restriction-ligation of HSVd cDNA fragments to pMD201t. 1 ul of the reaction was transformed into DH5-Alpha electro competent cells and 1/5 was plated onto kanamycin agar plates. **B)** Ligation of ELVd cDNA fragments to pMD201t. 2 ul of the reaction were transformed into DH5-Alpha electro competent cell and plated onto kanamycin agar plates. **C)** Control of BsaI-digested pMD201t. 50 ng of of pMD201t digested with BSAI were transformed into DH5-Alpha electro competent cells and plated onto kanamycin agar plates. **D)** Control of restriction-ligation of pMD201t. 1 ul of the reaction was transformed into DH5-Alpha electrocompetents cells and plated onto kanamycin agar plates.

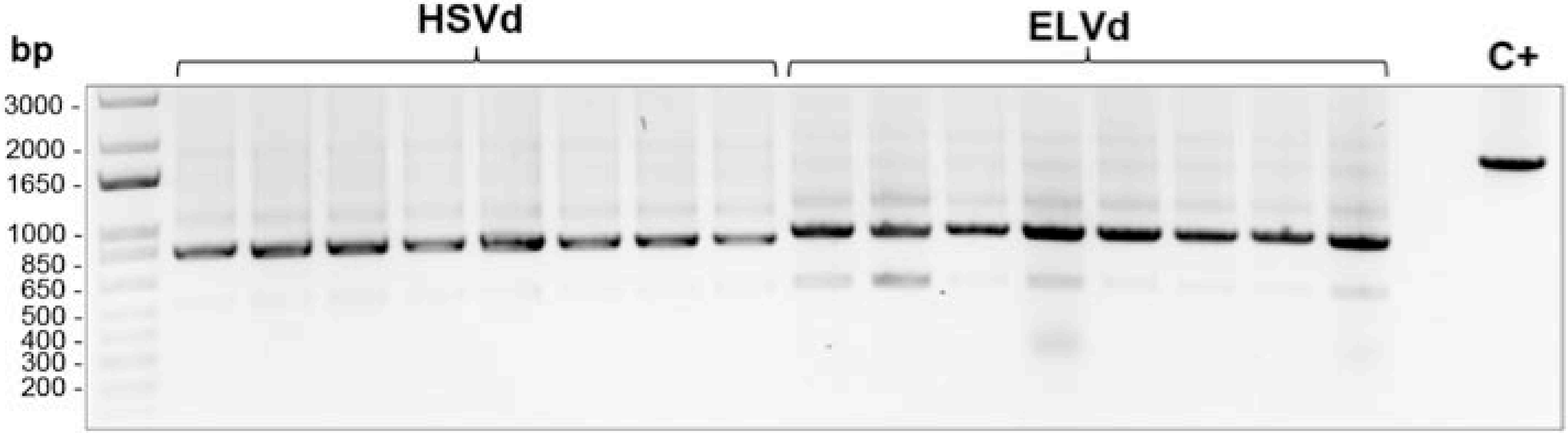

Additional file 2: Figure S2. Colony PCR of pMD201t. Agarose gel 1% showing PCR amplification of eight individual colonies of pMD201t ligation of HDVd fragments (left) or ELVd fragment (right). The observed size correspond to the viroid dimer plus part of the terminator and promoter sequence. A positive control of PCR amplification (C+) was the undigested vector.

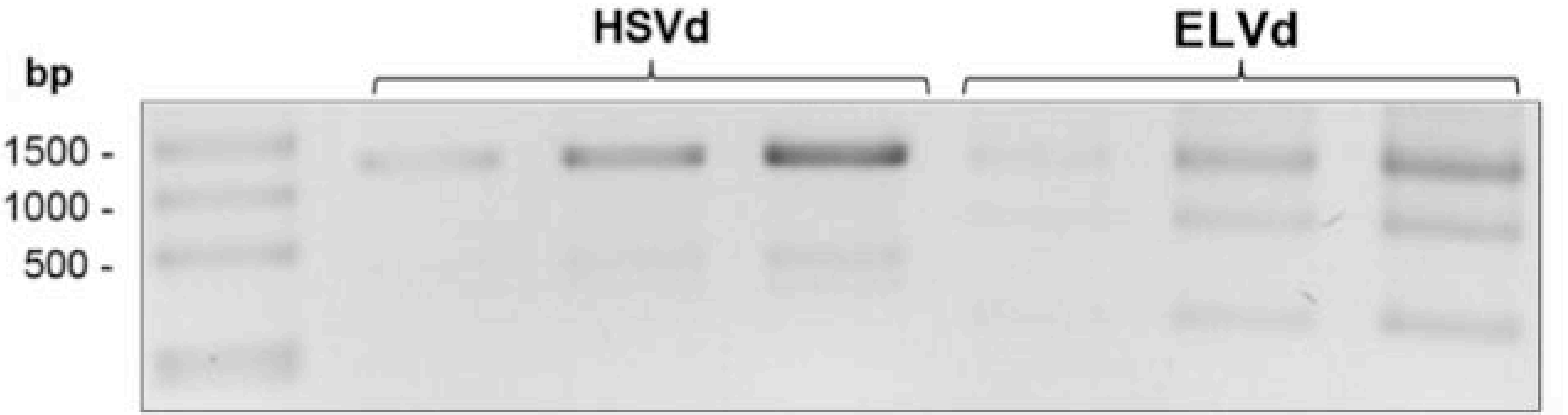

Additional file 3: Figure S3. *In vitro* transcription of pMD201t. 400 ng of linearized pMD201t HSVd/ELVd was transcribed with T7 RNA polymerase. Serial dilutions of linearized pMD201-HSVd transcription (left) and linearized pMD201t-ELVd (right). RiboRuler High Range RNA Ladder (Thermo Fisher Scientific) was used to estimate the RNA concentration, as for the loaded volume (0,83 *u*l) each ladder band corresponds to 50 ng.
