# Supplementary material for: Highly efficient construction of infectious viroid-derived clones
Source: Plant Methods. 2019 Aug 1;15:87. doi: 10.1186/s13007-019-0470-4 (PMC6670230; doi:10.1186/s13007-019-0470-4)
Supplement: Supplementary file 3 — Additional file 3. In vitro transcription of viroid RNA from pMD201t-HSVd and pMD201t-ELVd constructs. [file 13007_2019_470_MOESM3_ESM.pdf]

**Additional file 3: Table S2:** Details of the viroid related sequences containing the restriction sites used in our method. As can be observed, only the 0,19% of the known viroid variants in both *Pospiviroidae* and *Avsunviroidae* families contain both restriction sites. Viroid sequences were recovered from https://www.ncbi.nlm.nih.gov/nuccore.

| | **Number** | **Percentage** |
|---|---|---|
| **Viroid related sequences** | 11395 | 100% |
| **Containing BsaI sites** | 645 | 5,66% |
| **Containing BsmBI sites** | 503 | 4,41% |
| **Containing both BsaI and BsmBI sites** | 22 | 0,19% |
